# The Extent, Causes, and Prevention of Premature Death

**Published:** 1885-06

**Authors:** Eugene Foster

**Affiliations:** President, Augusta, Georgia


VOL. II. JUNE, 1885. NO. 4.

### Original Communications.

### THE EXTENT, CAUSES AND PREVENTION OF PREMATURE DEATH.

AN ADDRESS DELIVERED BEFORE THE MEDICAL ASSOCIATION OF GEORGIA, AT ITS THIRTY-SIXTH ANNUAL SESSION, IN SAVANNAH, APRIL 15TH, 1885, BY EUGENE FOSTER, M. D., PRESIDENT, AUGUSTA, GEORGIA.

*Fellow-Members of the Medical Association of Georgia:*

Section II, Article 5 of the Constitution of this Association declares it to "be the duty of the President to prepare and deliver, at the opening of the annual session, an address on some subject connected with the interests and objects of this Association."

I will, therefore, avail myself of this opportunity, which your courtesy has assigned me, to present for your thoughtful consideration a subject, which seems wholly unknown to the laity, which receives only contempt or indifference from our law-makers, and the magnitude of which is unappreciated by the vast majority of general practitioners of medicine in America.

In the limited time at command I will not attempt to discuss the subject in all its bearings upon man as an individual and upon society. I invite your attention to

THE EXTENT, CAUSES AND PREVENTION OF PREMATURE DEATH.

I define premature death to mean every death from any cause, save old age, or the natural decay of the structures and functions of the body.

What is the normal, inherent longevity of man? Answer this question and we establish the standard by which to determine what is premature death. The most eminent writers on this subject have from observation, anatomical and physiological data established the fact that the natural, inherent life of man is from eighty to one hundred years. Every human being has a natural right to this length of life. Every death at an age short of it is unnatural, and attributable to man's ignorance or carelessness.

Every human being should, in the language of Job, "come thy grave in a full age, like as a shock of corn cometh in his season." So much for man's inherent capacity for longevity. What is the length of life attained in the United States? By the census of 1870 the following facts were ascertained: The total mortality for the year was 492,263. Of this number 110,445 died before they had reached one year of age; 203,213 under five years; 16,000 died between the ages of 45 to 50; 13,246 died between the ages of 55 to 60; 14,000 died between the ages of 65 to 70; 11,000 died between the ages of 75 to 85; 4,500 died between the ages of 85 to 90; 1,300 attained to 95 years and upwards. Of the whole number of decedents, 17,429 lived to 80 years and upwards—in other words, 3.5 in every 100.

An examination of the Compendium of the United States Census for 1880 shows the following facts: The total mortality for the year was 756,893. Of these, 175,266 died under one year of age and 302,806 died under five years of age.

Have your minds followed me in this recital of facts? If so, you have detected a most amazing and unwarrantable loss of human life. What a spectacle! Vast numbers of children dead before the first anniversary of their birthday. Thousands upon thousands of precious, helpless infant lives yearly offered as holocausts upon the altar of man's ignorance or carelessness. These tables show only the actual loss of life. They do not tell, except indirectly, the appalling amount of pain and suffering which racks man in soul, body and estate. They do not disclose the deformity of body, nor the moral and physical decrepitude of a race so careless of that greatest of blessings—life.

What are the causes of this sacrifice of human life?

There are 1,146 diseases which affect mankind and require the study and attention of the physician. Of this number 58 are general diseases—*i. e.*, diseases affecting the whole system. These are divisible into the two following classes: 1st. Those which are infectious or contagious—33 in number; 25 other diseases—including consumption, scrofula, rheumatism, cancer, etc., make up this class. There are 1,080 affections classified as local diseases—*i. e.*, diseases having a localized seat in some organ or part rather than in the body altogether. The remainder have been classified as "other conditions of disease." These are not necessarily associated with general or local diseases—including "premature birth," "old age," "debility," "general exhaustion of all the organs of the body without specific disease," etc., etc.

An examination of the mortuary statistics of the United States Census of 1870 shows that 27 diseases caused nearly 75 per cent. of the total mortality. Every intelligent physician will admit that by the application of the well-established principles of sanitary science, man has absolute power to either wholly prevent or mitigate every one of these diseases. Examine these mortuary statistics for yourselves. Place in one column those which are known to be preventable, and in another column those known not to be preventable, and I think that even you—intelligent physicians daily ministering to the sick and suffering sons of Adam—will be surprised at the mortality from avoidable diseases, as compared with that from the non-avoidable.

The following table shows the deaths recorded from the 27 diseases referred to:

### U. S. CENSUS 1870.

### CLASS I—GENERAL DISEASES—DEATHS FROM CONTAGIOUS AND INFECTIOUS DISEASES.

| | |
|---|---|
| 1. Smallpox | 4,507 |
| 2. Measles | 9,237 |
| 3. Scarlet Fever | 20,320 |
| 4. Typhus Fever | 1,770 |
| 5. Enteric (Typhoid) Fever | 22,187 |
| 6. Malarial Fevers | 11,683 |
| 7. Diphtheria | 6,303 |
| 8. Whooping Cough | 9,008 |
| 9. Erysipelas | 3,162 |
| Total deaths from nine diseases of class I. | 88,177 |

### CLASS II—GENERAL DISEASES—NON-CONTAGIOUS.

| | |
|---|---|
| 1. Rheumatism | 2,912 |
| 2. Cancer | 6,224 |
| 3. Scrofula | 3,418 |
| 4. Consumption | 69,896 |
| Total deaths from four diseases of class II. | 82,450 |

### CLASS III—LOCAL DISEASES—DISEASES OF THE NERVOUS SYSTEM.

| | |
|---|---|
| 1. Encephalitis | 13,701 |
| 2. Meningitis | 3,334 |
| 3. Hydrocephalus | 4,041 |
| 4. Epilepsy | 1,414 |
| 5. Convulsions | 12,751 |
| Total deaths from five diseases of class III. | 35,241 |

### CLASS IV—DISEASES OF THE RESPIRATORY SYSTEM.

| | |
|---|---|
| 1. Croup | 10,692 |
| 2. Bronchitis | 4,049 |
| 3. Pneumonia | 40,012 |
| 4. Pleurisy | 1,084 |
| Total deaths from four diseases of class IV. | 55,837 |

### CLASS V—DISEASES OF THE DIGESTIVE SYSTEM.

| | |
|---|---|
| 1. Teething | 3,247 |
| 2. Enteritis | 9,046 |
| 3. Dysentery | 7,912 |
| 4. Diarrhœa | 14,195 |
| 5. Cholera infantum | 20,255 |
| Total deaths from five diseases of class V. | 54,655 |

A brief examination of the known facts of the principal diseases figuring in the death records will enable us to determine if or not they can be prevented or mitigated. First, take small-pox, measles, scarlet fever, whooping cough and diphtheria. We find that these five affections caused, in 1870, 49,375 deaths. However they began, or from what sources originated, it is not our purpose to discuss on the present occasion. It is sufficient to know that at the present time they never appear among mankind except as the result of exposure of one individual to another sick of the disease, or from contact with infected lodgings, bedding, clothing, etc. Isolate the sick and disinfect all infected materials and houses used by them and it will be impossible for either of these affections to prevail in a community, no matter how thickly populated. This is the medical opinion in all countries. It is true that a few learned physicians claim that diphtheria and scarlet fever may also originate from insanitary surroundings. But these gentlemen affirm that these diseases may be prevented by strict medical police. Under either theory of causation they are wholly preventable. In relation to small-pox, it is proper to add that vaccination and re-vaccination, generally and systematically performed, will prevent development of small-pox. If preventive medicine has demonstrated anything, it is, that by enforcing the above named measures you can stamp out every one of these deadly diseases.

### TYPHOID FEVER.

This disease caused 22,187 deaths. Listen to what John Simon, the greatest sanitarian in the world, says of it: "I would say that it is difficult to conceive, in regard to any causation of disease in a civilized community, any physical picture more loathsome than that which is here suggested; that apparently, of all the diseases which are attributable to filth, this, as an administrative scandal, may be proclaimed as the very type and quintessence; that, though sometimes by covert processes, which I will hereafter explain, yet, far oftener, in the most glaring way, it apparently has an invariable source in that which of filth is the filthiest; that apparently its infection runs its course, as with successive inoculations from man to man, by instrumentality of the molecules of excrement, which man's filthiness lets mingle with his air and food and drink."

### MALARIAL FEVERS.

This class of diseases is the product of heat, moisture and vegetable decomposition in the soil. These fevers are preventable by thorough drainage of the soil. They were formerly as prevalent in England as America, but thorough drainage has almost wholly banished them from England. In America we are absolutely indifferent to 11,683 deaths yearly from this preventable disease.

### SCROFULA

Is in the vast majority of instances a disease hereditarily acquired. Its causes mainly lie beyond the conception of the infant, and preventive measures will be discussed under marriage of unhealthful individuals.

### CONSUMPTION.

This, of all the diseases affecting mankind, is the most general in prevalence and fatal in results. It is caused by hereditary transmission, foul air, unhealthful pursuits, damp soils, syphilis and alcoholism. Preventive measures will be discussed during the progress of this paper. The annual deaths from this disease reach the enormous number of 69,876.

### EPILEPSY.

In the opinion of high medical authorities, 60 per cent. of the cases of epilepsy are due to heredity.

### CONVULSIONS.

Of the 12,751 deaths recorded from convulsions, 8,563 were children under one year old. Generally speaking, this is a symptom of disease—not a disease in itself. The causes are most generally febrile diseases, hereditary transmission of nervous constitutions, errors of diet, etc.

### HYDROCEPHALUS.

In the vast majority of cases of this affection, the cause is traceable to transmission of strumous diathesis from parent to child.

DIARRHŒAL DISEASES.

Simon truly says of this class of diseases: "In all filthy districts, one particular class of diseases seems specially apt to stand in relief, the diseases, viz., which, in respect of their leading symptom, may be generalized as diarrhœal. These diseases, in their relation to filth, deserve very special attention. First, on their own account, as extremely large causes of death, etc. * * * The mucous membrane of the intestinal canal seems to peculiarly bear the stress of all accidental putridities which enter the blood. Whether they have been breathed, or drank, or eaten, or sucked up in the blood vessels from the surface of foul sores, or directly injected into the blood vessels by the physiological experimenter, there peculiarly the effect may be looked for; just as wine, however administered, would get into the head, so septic ferment, whencesoever it may have entered the blood, is apt to find its way thence to the bowels, and there, as universal result, to produce diarrhœa." The United States Census shows a yearly death of more than 50,000 persons from this class of diseases—24,000 of them infants, under one year of age. In considering diarrhœal diseases of infants, it must be remembered that artificial feeding is a powerful factor in their production.

THE DISEASES OF THE RESPIRATORY SYSTEM, including croup, bronchitis, pneumonia and pleurisy, produced 55,837 deaths. The principal causes of this class of affections are exposure to cold and residence in a damp locality.

I leave to your leisure the further study of the details of the diseases considered above.

There are mainly four essentials to the attainment of the inherent, potential longevity of man as a race. They are:

1. Purity of atmosphere and water.
2. Wholesome food, both in quantity and quality.
3. The inheritance of a healthful constitution.
4. Freedom from contagious and infectious diseases.

Man in his individual efforts has little or no control over either of these essentials of life. The only power which secures the individual the enjoyment of either of these blessings is the State.

In all societies the State is the delegated guardian of man's life, as well as his liberty and property. This is the idea which I wish to elaborate this morning.

First, can an individual protect himself against pollutions of the air he breathes or the water he drinks? Take any city you please and examine the sources of pollutions of air and water and you will find a want of drainage and a failure upon the part of the municipality to thoroughly and rapidly remove all refuse matters—both liquids and solids—from the inhabited area. There is one fact which has been demonstrated in the history of every nation on the globe, and to which the science of medicine in all ages and countries testifies: that fact is that no population living in the midst of filth, or within reach of its contaminating influences, has ever been healthy, or even free from recurrences of pestilential diseases, and that such peoples have succumbed to the various diseases known to be caused by these influences; whereas, under opposite conditions, other communities have remained comparatively or absolutely free from them—the immunity being in direct ratio with the absence of the former surroundings. This is a self-evident and undeniable truth which it seems absurd to think it necessary to state. But what, from a sanitary standpoint, is to be considered filth? Any putrefiable substance—animal or vegetable—which is allowed to remain within or near an inhabited area, is filth, and in direct ratio with amount of these putrescent substances will be the unhealthfulness of the population. If this be so (and who denies it?) why should we be amazed at the extent of preventable sickness and death in America? The medical officer of the Privy Council of England, in showing the insanitary condition of his own country, said:

"There are houses, there are groups of houses, there are whole villages, there are considerable sections of towns, there are even entire, and not small towns, where general slovenliness in everything which relates to the removal of refuse matter. Slovenliness, which in very many cases amounts to utter bestiality of neglect, is the local habit; where, within or just outside each house, or in spaces common to many houses, lies for an indefinite time, undergoing fœtid decomposition, more or less of the putrefiable refuse which house life, and some sorts of trade life, produce; excrement of man and brute, and garbage of all sorts and ponded slop waters sometimes lying bare on the common surface; sometimes unintentionally stored out of sight and recollection in drains or sewers, which cannot carry them away; sometimes held in receptacles specially provided to favor accumulation, as privy pits and other cesspools for excrement and slop-water, and so called dust-bins receiving kitchen refuse and other filth. And with this state of things, be it on large or small scale, two chief sorts of danger to life arise: one, that volatile effluvia from the refuse that pollute the surrounding air, and everything which it contains; the other, that the liquid parts of the refuse pass by soakage or leakage into the surrounding soil to mingle there, of course, in whatever water the soil yields, and in cases thus to occasion the deadliest pollution of wells and springs. To a really immense extent; to an extent indeed which persons unpracticed in sanitary inspection could scarcely find themselves able to imagine, dangers of these two sorts are prevailing throughout the length and breadth of this country, not only in their slighter degrees, but in degrees which are gross and scandalous, and very often, I repeat, truly bestial. And I state all this in unequivocal language, because I feel that, if the new sanitary organization of this country is to fulfill its purpose, the administrators, local and central, must begin by fully recognizing the real state of the case, and with consciousness that in many instances they will have to introduce for the first time, as into savage life, the rudiments of sanitary civilization.

A second point, which equally with the above needs to be recognized by all who are responsible for the prevention of filth diseases is: that filth does not only infect where it stands, but can transmit its infective power afar by certain appropriate channels of conveyance; that, for instance, houses which have underground drainage communication with cesspools or sewers may receive through such communication the same filth-infections as if excrement stood rotting within their walls; and that public or private water reservoirs, or water conduits, thus giving accidental admission to filth, will carry the infection of the filth whithersoever their outflow reaches. Thus it has again and again happened that an individual house, with every apparent cleanliness and luxury, has received the contagion of enteric (typhoid) fever through some one unguarded drain inlet; or that members of such houses have simultaneously received the infection, as an epidemic, in places where the drain inlets in general have been subjected to undue air pressure from within the sewer. And thus equally on the other hand it has again and again happened that households, while themselves without sanitary reproach, have received the contagion of enteric (typhoid) fever through some nastiness affecting (perhaps at a considerable distance) the common water supply of the district in which they are.

If this state of things exists in England—where the Queen, the mighty Premier, most learned statesmen, lawyers, ministers and physicians are united as a band of brothers in attempting to lighten the burdens of sickness, sorrow and death of the meanest of her subjects—what can we say of America, where the philanthropist who turns his attention and efforts to public health is regarded as an idiot unfit even for association with the inmates of the lunatic asylums, and worthy of only scorn or contempt of the so-called educated classes?

There is no proposition better demonstrated by common observation and by scientific medicine than the fact that man cannot live upon an undrained soil and remain free from the deadly prevalence of malarial fever, rheumatism, pneumonia, bronchitis, croup, diarrhœal diseases, typhoid fever, consumption, etc. Show me in this section of country a thoroughly drained city. You cannot do it. It does not exist. But some one will ask, what has undrained soil to do with consumption? To answer this question it will be well to assume the yankee method—ask a question in reply. Why does New Orleans, with all the other climatic advantages, etc., show a higher death rate from consumption than any other city in America? If you will go down to New Orleans and put a spade into the soil, you will find the ground water-logged. Soil water in many sections of the city can be found within about eighteen inches of the street service. The influence of soil moisture in the production of consumption is no theory—it is a demonstrated fact. It has been demonstrated by Dr. Bowditch, of Massachusetts, and Dr. Buchanan, of England, that soil moisture and soil pollution are directly causative of this disease, as well as many other fermentative zymotic diseases. The following table shows the results of sewerage and drainage in portions of England as quoted by Baldwin Latham's Sanitary Engineering:

| NAME OF TOWN. | Average mortality per 1,000 prior to construction of works. | Average mortality per 1,000 since completion of works. | Saving of life, per cent. | Reduction of typhoid fever rate, per cent. | Reduction in rate of Phthisis Pulmonalis, per cent. |
|---|---|---|---|---|---|
| Banbury . . . | 23.4 | 20.5 | 12 | 48 | 41 |
| Cardiff . . . . | 33.2 | 22.6 | 32 | 40 | 17 |
| Croydon . . . | 23.7 | 18.6 | 22 | 63 | 17 |
| Dover . . . . | 22.6 | 20.9 | 7 | 36 | 20 |
| Ely . . . . . | 23.9 | 20.5 | 14 | 56 | 47 |
| Leicester . . . | 26.4 | 25.2 | 4½ | 48 | 32 |
| Macclesfield . . | 29.8 | 27.3 | 20 | 48 | 31 |
| Merthyr . . . | 33.2 | 26.2 | 18 | 60 | 11 |
| Newport . . . | 31.8 | 21.6 | 32 | 36 | 32 |
| Rugby . . . . | 19.1 | 18.6 | 2½ | 10 | 43 |
| Salisbury . . . | 27.5 | 21.9 | 20 | 75 | 49 |
| Warwick . . . | 22.7 | 21.0 | 7½ | 52 | 19 |

From the above table it will be seen how remarkable was the reduction of mortality from consumption, varying from 11 to 49 per cent. These observations extended over a number of years.

This table also shows the reduction of typhoid fever to be from 10 to 75 per cent. Study this table. It speaks for itself.

It is unnecessary to give further illustrations of the beneficial effects of partial or thorough sewerage and drainage upon the health of inhabitants of various communities. These benefits have been demonstrated in hundreds of instances. The records of preventive medicine literally swarm with them.

If you admit the baneful effects of an undrained soil upon the healthfulness of a city, what must be the unhealthful influences upon rural populations by residence near overflowed, undrained and swamp lands? Take the overflowed lands in the States along the Atlantic and Gulf coasts. Here is an extract from a report of the Superintendent of the United States Coast Survey:

| Name of State. | Extent of overflowed lands. |
|---|---|
| Maine | 12 square miles |
| New Hampshire | 10 square miles |
| Massachusetts | 46 square miles |
| Rhode Island | 25 square miles |
| New York | 86 square miles |
| New Jersey | 360 square miles |
| Pennsylvania | 11 square miles |
| Delaware | 88 square miles |
| Maryland | 210 square miles |
| Virginia | 500 square miles |
| North Carolina | 3,540 square miles |
| South Carolina | 2,400 square miles |
| Georgia | 1,375 square miles |
| Florida | 18,422 square miles |
| Alabama | 750 square miles |
| Louisiana | 17,718 square miles |
| Mississippi | 4,798 square miles |
| Texas | 8,800 square miles |
| Total | 59,156 square miles |

Enormous as is this aggregate of overflowed lands, it must be remembered that this estimate only shows the extent of this class of lands directly communicating with the tide. It does not include enormous bodies of swamp lands in the interior of many of these States.

To bring the question home take the State of Georgia. What extent of territory is covered by these overflowed lands? Ten years ago Mr. P. W. Alexander, Secretary of the Executive Department, stated that "the swamp, water-soaked and overflowed lands of Georgia equal in extent probably the area of the Kingdom of the Netherlands (Holland.") If this estimate be correct, the total area of undrained lands in our State reaches the enormous amount of 12,731 square miles. The total area of Georgia is 58,000 square miles.

Do you wonder that malarial fevers run riot over the people of the State, making them sallow, weakly, unhealthful, paralyzed in energy, and killing them by scores with this preventable disease, which is far more deadly in its effects upon our people than yellow fever and cholera combined? Here is not only a waste of productive energy, health and life, but the reclamation of these lands would add thousands and hundreds of thousands of acres of richest lands to your cultivatable area, which, under intelligent tillage, would afford sustenance for a population three times as large as that of the State of Georgia at the present time.

FOOD

Must be nutritious in quality and sufficient in quantity. For all purposes of food supply the human body is like the steam engine —the food man eats is intended to serve as fuel from which the mechanism of the stomach is to evolve muscular force, heat, thought, etc., and, beyond the power of the steam engine, to lay by a store of force for use when digestion is deranged. The healthful body must be maintained in equilibrium—*i. e.*, the constructive and destructive forces must be made to balance. If the destructive processes exceed the constructive, there is a lowering of vital force in direct ratio with the disturbed harmonies. If the constructive largely overbalance the destructive processes, unhealthfulness by fatty degenerations of the liver, kidneys, heart, etc., will be the result. There is a lamentable want of information upon the part of the people as to the various kinds of food for man, their dietetic value, the relations which food bears to health and labor, and upon the physiology of digestion. It should be one of the most solemn duties of government to impart such information to the populace. The muscular strength of the people is primarily the source of the prosperity of a nation, and this can only be secured by the proper use and distribution of food. If a few individuals suffer for food the detriment to the State is little; if from any cause scarcity of food prevails in a nation, the productive energy is impaired, and dire pestilential diseases, such as scurvy and relapsing fever, will decimate the inhabitants. Man is an omniverous animal and may secure food for sustenance of full bodily vigor from either the products of the water, the forests or the soil. His main dependence for food is upon cereal crops, and if these are blighted famine with its concurrent diseases ensue. In this age of steam on water and land man's philanthropy will respond to distress of the farthest nation of the earth, and the day of famine should be at an end. The most important question in relation to food supply in relation to premature death is the proper nourishment of infants. The mother's breast is the generous, sympathetic fount from which should flow the nourishment for her babe. It has become fashionable in some circles of society for the mother to shrink from and shirk the high and solemn duty of nursing her own infant; a diseased or filthy negro, or a half-starved cow fed on swill, is inducted into the office of feeding the child, and when it is well-nigh starved to death it is fed on farina, infants' food, etc. This species of moral depravity and inhumanity is lower than the instinct of the brute, which always tenderly nurses and defends her little one. It was regarded as disgraceful in the highest degree for the Roman mother to neglect to suckle her own child, and put it to the breast of a nurse. May the day speedily come when the American mother shall appreciate this high and sacred trust.

### THE INHERITANCE OF A HEALTHFUL CONSTITUTION.

To secure healthy progeny the parents must be healthful. This is a proposition so plain that even the fool is compelled to assent to it, and yet does man pay any attention to this inexorable law of nature? Do you not constantly meet with the most glaring instances of man's failure to appreciate this solemn truth? Do you not frequently observe individuals who are diseased well-nigh unto death with consumption, scrofula, syphilis, epilepsy, etc., leading pure and lovely women to the marriage altar? These men are liable to infect the wife, and will almost certainly transmit their disease to their offspring, and will live about long enough to beget a few sickly, degenerate children and die, usually leaving to the children as their sole patrimony the diseased constitution which carried the father to the grave. How often do you see women burdened almost unto death with diseased bodies eagerly entering the wedded estate? In this age we hear much of fine strains of Jersey cattle, Southdown sheep and blooded horses, and but little of the physical perfection of human beings. When a young woman is courted by one of your society snobs, who has only a few months or years to live, did you ever hear her parents ask what is this man's health? Never. The questions propounded are: What is his social standing? How much money is he possessed of? What is money in comparison with the ruddy glow and the productive energy of health? What is the value of social position when its possessor is a corrupting mass of disease? He may be a Crœsus and expect to purchase pleasure with his gold, but when health is gone he has no capacity for its enjoyment. He may be of great social prominence, and yet when the mask is removed and his disgusting deformity of disease is laid bare, it sends a chill of horror over the heart of the disappointed and unhappy wife. Every day, in all sections of this country, scores upon scores of infants are born into the world inoculated with scrofula, syphilis, consumption, epilepsy, chorea, insanity, or the alcoholic diathesis, and this often, to a frightful extent, in the highest society of the nation. What can society expect from marriage of diseased persons? The children of a large proportion of these unions drop dead from their mother's womb, or perish soon after birth, or, if they survive, they are in a state of primitive decadence, and a burden to relatives, friends or the community. No man who is unhealthy has the moral right to assume the royal station of father. No woman with scrofula, consumption, etc., has the right to aspire to the high and sanctified office of motherhood. If the law of the land is not and cannot be made powerful for prevention of marriage of such diseased individuals, is it asking too much of a wholesome and enlightened public sentiment to frown upon such wrongs to society? But we are assured that all efforts to protect children from inherited diseases will produce greater evil than good; that an individual marrying, with a strong taint of insanity, liable to bring into the world a whole family of lunatics, should be untrammeled to do so for the reason that such marriages often produce whole families of genuises. If this is the process necessary to the manufacture of genius, may God Almighty save America from the production of a genius.

4th. Man's stupidity in permitting cases of dangerous contagious diseases to scatter abroad the seeds of their infection when he is all powerful to control them.

Truly has it been said, "the curse causeless shall not come." These diseases, in their origin and diffusion, are governed by inexorable laws, and while the chemist and the microscopist may be unable to detect their essence, yet the laws of their rise and spread are definitely known, so that man can either prevent or mitigate them if he will but do so. If he fails to exert his controlling power over these affections, either through systematic ignorance or indifference, the danger to mankind is far greater in this age of advanced civilization than even amid the gloom and desolation of the dark ages. In this day of rapid transit the facilities for diffusion of contagious diseases are such as to eminently threaten the welfare of every community. All of this class of diseases possess the property of portability. A family may imbibe the infection of either of this class of affections and traverse the whole Union before the disease declares itself. By infection of wearing apparel, bedding, etc., the germs of these diseases may be transported from continent to continent, and scatter their infection wherever infected materials are carried.

No country on the face of the earth is so imminently threatened with devastation from portable diseases as America. The immigration to this country of people from all lands on the globe is amazing in extent, and unprecedented in the history of any other nation. Into this land constantly pours a great gulf-stream of humanity, composed largely of the restless, dissatisfied, turbuent, poverty-stricken and oppressed people of all climes and countries. Day after day, week after week, enormous vessels filled with human freight discharge their living cargoes into our seaports, and hurriedly put back to foreign lands for another burden of human beings seeking a home in the new world. From 300,000 to 1,000,000 immigrants land upon our shores every year. Go into Castle Garden and listen to the jargon of these new citizens, and the various languages suggest the possibility of an attempt to build anew the Tower of Babel. Look at these new-made sovereigns, ragged, filthy and penniless, their little bundles packed amid the squalor and diseases of the dangerous homes from which they emigrated. Into the great harbors of New York, Philadelphia, New Orleans and San Francisco these millions of peoples are hastily thrown upon the wharves, and by hundreds of fast boats are carried over thousands of miles of rivers, or over the 121,000 miles of railways, and scattered into every community in America, and with them are carried the infectious diseases of the places from whence they came. If the United States government fails to exercise a rigid sanitary surveillance over these peoples, both at the ports of departure and upon arrival in our harbors before they are admitted to the docks, what rational hope can we entertain of security against weekly importations of deadly infectious and contagious diseases? Does the General government, or any State government, operate such service upon an adequate scale? Where is the man who is so brazen-faced in uttering falsehood as to answer Yes? By this failure of the National government the commercial stability, and the peace, prosperity and happiness of every community are daily jeopardized. Thus are these diseases readily brought into our cities, and as soon as one is driven out another stares you in the face. The only hope of protection in this day is for the municipality to keep skilled and faithful guardians on the alert to meet and conquer these direful affections.

What value do we place upon human life when every year five diseases—small-pox, measles, scarlet-fever, diphthéria and whooping-cough—kill fifty thousand inhabitants of America? Every one of these diseases is wholly preventable, and yet the people have never arisen in their might and majesty and declared that these things of death shall no more occur. The most appalling feature of this problem is the fact that fully thirty-five thousand of these deaths are in children under five years of age.

If the American mind can only be reached through lessons impressed by cholera and yellow fever, we may profitably take these two from which to draw illustrations of the whole class now under consideration.

Why should cholera be found in America? This is not its home. It cannot be kept alive here except by annual importation from foreign lands. It is an exotic disease, endemic chiefly in the valley of the Ganges. It is brought into this land by the great white sails of commerce. Whenever it prevails in Europe it is almost certain to come as an unwelcome visitor among us. Entering our great seaport towns, it is rapidly carried by steamboats and railroad cars into the numerous towns and cities located on these channels of trade. The natural history governing the rise and diffusion of cholera is well known, and by definite measures known to the sanitarian it can be kept out of our country, and even stamped out if by chance it finds a lodgment here. Yet, notwithstanding our power to stamp out this frightful disease, this mighty nation, indifferent to either the voice of humanity or commercial prosperity, did nothing to protect our people, and in 1873 it invaded thirteen States and fastened its remorseless fangs into the heart of more than two hundred towns and cities.

Are we powerless against yellow fever? By no means. Man has absolute power to prevent it. Its existence in this country is a shame and a disgrace to the civilization of the age. Look at Boston. Prior to 1805 epidemics of yellow fever prevailed there, but the disease has been banished from that city by the rigid administration of sanitary laws, and no epidemic of this disease has visited Boston since 1805. The same is true of Providence, Rhode Island. Philadelphia had seventeen epidemics of yellow fever prior to 1854. But the sanitary government has kept the city free from 1854 to this date. Look at New York. Prior to 1822 epidemics of yellow fever ran through the city and in some years murdered her citizens by thousands. Notwithstanding the wonderful growth and density of the population of New York, the sanitary bureau has banished the disease from the city. No epidemic of yellow fever has prevailed in the metropolis since 1822. This absence is not due to want of facilities for importation of the disease, for the excellent and accomplished Health Officer of the port says that not a year passes but what cases of yellow fever are taken from the shipping to the quarantine station. What these cities have done, every other city in America can do. Yellow fever in America is an administrative scandal.

I have not attempted to follow the subject of this address into all of its details. I have contented myself with an effort to show that in the vast majority of diseases causing premature death in America, man, by the application of the well-established principles of hygiene, has arbitrary power to prevent or control them. Nothing is necessary to produce these results but to arouse the people to an appreciation of the sacredness of human life, and the possibilities of life under intelligent administration of public and private hygiene.

The average American citizen only dreads the pestilence that walketh in darkness, and the destruction that wasteth at noonday. If cholera or yellow fever appears in a city in America, the whole country becomes alarmed; but we Americans are absolutely indifferent to other preventable diseases which yearly produce one hundred times greater mortality than cholera and yellow fever combined. I affirm, deliberately, that these latter diseases, which so alarm and horrify the inhabitants of America, are insignificant in death-dealing when compared with the mortality from diseases over which man has absolute powers of control, and which he makes no effort to prevent or mitigate. Is this statement true? In 1873, epidemics of cholera and yellow fever ran through the country and produced alarm in every city in the land; commerce with the infected cities was suspended; citizens refugeeing from homes, and penned up by shot-gun quarantines; business in the afflicted cities paralyzed; communities pauperized; the public press of the nation teeming with telegrams telling of the harvests of death being gathered in these communities; express companies were burdened with provisions, clothing and medical supplies; money by thousands and hundreds of thousands of dollars was sent from all sections, and physicians and nurses by scores hastened to the rescue of the afflicted peoples. All eyes were directed and hearts opened to the cry of distress; and yet, what was the total mortality from both yellow fever and cholera? 8,000. In the same year fully 12,000 Americans died of malarial fever; 20,000 from scarlet fever; 22,000 from typhoid fever; 50,000 from dysentery, diarrhœa and cholera infantum; 70,000 from consumption; 9,000 from measles, and 9,000 from whooping cough; but who heard anything of these diseases? Did the people of this nation feel any alarm? Was money expended to prevent or mitigate the ravages of these diseases? Was trade or intercourse suspended? Did Congress send a commission of experts to investigate the causes and means of prevention, as they did of the epidemics of cholera and yellow fever? No. The Congress as well as the people ignored everything but the yellow fever and cholera, and organized a national board of health, instructed to quarantine against these two diseases. Congress and the people then breathed a sigh of relief and dismissed the subject from further consideration. Is life less sacred because sacrificed by malaria, tyhoid fever, measles or scarlet fever, than from cholera or yellow fever? It is true that the havoc of cholera and yellow fever was far more rapid in destruction, but these diseases rarely afflict us more than once in a decade, while the others are always with us, reaping enormous harvests of death each year.

What value does society place upon human life where, as in America, one hundred and fifty thousand bar-rooms are licensed to sell accursed liquid fire? Think of the magnitude of this evil. Gather together these dens of infamy and destruction in America and place them into one street; allow twenty feet to each bar-room, and place them side by side on both sides of this avenue, and you will have a street of more than two hundred and sixty-five miles in length, in which every occupant is devoting his time and business to the destruction of his fellow-man.

Does any sane man question the fact that the use of intoxicating liquors at the present day is productive of poverty, crime, disease and death to an amazing extent? In the practice of your profession, do you not almost daily meet with wretched human beings diseased unto death from imbibing alcoholic drinks? Is not alcohol indirectly and directly causative of a large percentage of dyspepsia, skin diseases, functional disturbances and degenerative changes of the heart and blood vessels, the stomach, the lungs, liver, kidneys and nervous system?

I shall not attempt to follow the tracks of this fell-destroyer through all the various courses of disease. The distinguished Dr. B. W. Richardson, of London, shows that twenty diseases are caused by alcohol, and further says that alcohol led to one-fourteenth of the whole adult mortality of England and Wales. Dr. Hitchcock, President of the State Board of Health of Michigan, claims that 49,000 Americans die each year from the direct and indirect effects of alcohol. He shows the fearful percentage of idiotcy, insanity and criminals from this source, and closes by showing the money value of health and life sacrificed, the cost of maintenance of the sick, the idiots, insane, etc., the cost of funerals, etc., reaches the enormous sum of $200,000,000 each year. This estimate does not attempt to show the cost of the care of criminals, court processes, or pauperism, directly chargeable to alcohol. Yet this accursed traffic in human flesh and blood is made bold, and, in the eyes of some men, respectable by licensed permission of the majesty of the law of the land.

What value does government place upon health or life, when 200,000 courtesans are permitted to cumber the cities of America, and absolutely no effort made to suppress or control the evil, so that the youth of the land may be saved from ruin, or the infection of the brothel kept from the homes of spotless women and children. A few good and true men and women protest against the refusal of the civil authorities to attempt to suppress or control to the utmost limits possible this frightful social disorder; but what encouragement do these lovers of human welfare receive? Men speak in whispers of this great social evil, and ask that it be ignored, and tell you that you have no remedy, and therefore it is better to do nothing. Do nothing, indeed, when from 1,000,000 to 2,000,000 Americans are poisoned with syphilis from this source. In this Christian country, where the conversion of a few heathens to the cause of Christ is joyfully proclaimed from Sunday to Sunday from the pulpit, and where money by hundreds of thousands of dollars is expended for the cause of heathen missions, two hundred thousand fallen women are left to drift to death and hell, and to send out their infection into homes of purity and innocence, and we are told to do nothing!

With all these sources of disease, why should we be amazed at the extent of premature death in America ? With the air teeming with invisible poisons emanating from pollutions of soil which filthiness or carelessness lets loiter about him; with the water he drinks fouled with excrement; his system poisoned with alcohol, or the infection of the courtesan; the vast hereditary transmissions of sickness and degenerate constitutions; and the absolute indifference to prevention or control of contagious diseases by this animal fashioned in the image of his God, the fact that even fifty per cent. of human beings among decedents reach five years of age should amaze us.

What a comment! Man, "the crown of the visible creation; the last, the most complete, and the best finished production of the plastic power of Nature; the highest degree of its self-representation which our eyes are capable of seeing, or our senses of comprehending;" who, by the power of superior intelligence, lays tribute upon the birds of the air, the fishes of the sea, and the animal and vegetable productions of the land for his sustenance; endowed by nature with an inherent longevity far beyond all other animal existence, he is yet so careless of his own vital forces that in point of length of life he is but little above the brutes of the field.

In all ages of the world the prolongation of human life has engaged the study of the most learned of men, and as the result of that study an almost unbroken line of authorities, from the earliest dawn of civilization to the present time, may be cited, telling us that over the vast majority of diseases which produce premature death, man can exert arbitrary power to prevent or mitigate them. It is true that writers suggest various plans for securing this result, but they all unite in affirming that it is possible. Then why has man for thousands of years remained indifferent to preventable sickness and premature death ? The popular mind has ever failed to grasp the magnitude of the evil and the power of control. In savage as in civilized life man has been taught to believe that disease and death were imposed by God as manifestations of the divine wrath for violations of the moral law. As a consequence of this teaching, no systematic, general, intelligent, concert of action among peoples have ever been organized and operated upon a scale which the importance of the subject demands. An advancing civilization is constantly demonstrating that man is charging his own sins to the Almighty. Disease is a penalty for sin, but it is the sin of self-abuse. The vast majority of people, who assent to the proposition that man can ameliorate the elements of disease which surround him, insists upon each "toting his own skillet" in this warfare. It is true that in every civilized community at the present day there exists one or more sanitary organizations, but so far they have only been able to preach, and not enforce, the gospel of hygiene.

To widen the scope of human life, and promote the happiness and productive power of the race, is a field of work which should fire the zeal of the pure and good of every community. The health of the people is the wealth of a nation, and it is the highest and most sacred duty of government to spare neither time, talent nor money to advance the health of its citizens. Public health can only be advanced by organized effort. The organization must consist of municipal, State and national authorities, charged directly with this high and sacred trust.

The first organization must be municipal. No city can hope for healthfulness unless it has a properly organized and equipped health department. Its members should be chosen for the sole consideration of ability and integrity. This department should be charged with the duty of instructing citizens upon health problems, and clothed with authority to expend money judiciously to protect the municipal health, and to enforce, when necessary, sanitary laws.

The State should organize and sustain a health department in full accord with municipal organizations. This department should be equipped with money and talent to investigate the hidden sources of disease, and it should regulate the sanitary relations of municipalities, and preserve records of vital statistics, etc.

The nation should operate a central bureau of health, located at Washington, authorized to co-operate with municipal and State boards of health for the general welfare. The national health bureau should collect weekly, and, when necessary, daily reports of the prevalence of contagious and infectious diseases in the various cities and States, and inform threatened localities of the dangers environing them. The commerce of foreign nations seeking our shores should be supervised by sanitary consular service at the ports of departure and on arrival in our ports, and reports of contagious and infectious diseases therein promptly transmitted to all health authorities, and these diseases stamped out before vessels are admitted to our wharves.

If this be done upon a scale equal to the signal service bureau, the general government can keep cities and States daily informed of the progress of pestilential diseases, and, by sanitary signals, warn threatened communities to make preparation to meet and repel these public enemies. The national bureau should supervise inter-state commerce in its relation to health. This department should be fully equipped for investigation of disease germs, the laws of origin and diffusion of diseases, etc. Such a department could be so constituted and operated as to be financially economical, and be productive of human health, prosperity and happiness.

How can the sanitarian induce mankind to partake of the bounteous fruits of health and long life? I answer,

### EDUCATION.

Educate the children in public schools into an understanding of the elemental principles of anatomy, physiology and pathology. Teach the child the simple laws of health, and as he advances from grade to grade of the general school curriculum, let him be taught higher and higher grades of study of himself, so wonderfully and fearfully made. Teach him of digestion of food; the physiology of respiration; the composition and purposes of water; the laws of growth and decay; the causation and means of avoidance of the principal ones of the fatal diseases, etc., and you will find that as he arrives at each era of existence he will eagerly and persistently seek yet more knowledge of the high aims and possibilities of life. Then, as his reason and judgment expand, he will see that what appear to be self-denials for conservation of his vital forces will be known to be only the pleasurable and joyous avoidance of enemies ambushed to spring upon and crowd him down with poverty, suffering, disease and death.

Educate the legislator to understand that health is the capital of the nation. Let him sit at the feet of England's Premier (the mighty Beaconsfield) and hear him in one of the grandest speeches of his life declare, "The health of the people is really the foundation upon which all their happiness and all their power as a state depend. The health of the people is, in my opinion, therefore, the first duty of the statesman; and I am confident that there is no object of higher importance to engage the interests of society."

Educate the engineer in the relation of his work to hygiene. Let him learn that if his work be not skillfully executed, he is building beneath the earth's surface magazines of evil which shall, sooner or later, explode and scatter disease and death to the people whom he expected and desired to benefit.

Educate the architect as to the sanitary arrangement of dwellings, schools, churches, workshops, etc.

Educate the ministers of the land to an appreciation of the elements of sanitary civilization. Let him know that when the consumptive, the syphilitic or the leprous man or woman enters the holy bonds of matrimony, and the issues of such marriages die in infancy, and he is called upon to conduct the funeral services, that it is blasphemy to speak of such a death being a manifestation of God's loving care of the infant in removing it from future temptation and sin. When yellow fever or cholera is decimating a filthy community, let him not insult God by praying that he will cease to kill the people. To charge the Almighty with willful murder is to present him as a monster to be detested—not a Father to be adored. Such prayers issue from shameless ignorance or blasphemy. Better were it did such clergymen learn the lesson taught by the Premier of England, who, when cholera threatened the kingdom a few years since, and he was petitioned to appoint a day of fasting and prayer to induce the heavenly Father to save them from pestilence, declined to grant the petition, but replied, "Go home and see that your towns and cities are freed from those causes and sources of contagion which, if allowed to remain, will breed pestilence and be fruitful in death in spite of all the prayers of a united, but inactive people." Hasten the day when ministers of the gospel shall understand that the highest perfection of religion is only consonant with the healthy body. Let man live to old age, develop his faculties to the greatest perfection, day after day grow in knowledge and grace, and finally, when full of years, depart in glory. Let him understand that the prevalence of disease is under the reign of law, and that if man desires to be healthy he must not violate the laws of health. If the preacher thinks the sanitarian a fool, it would perhaps be well to remind him, who is all unobservant of the philosophy of events around him, that possibly Solomon in all his glory was as wise as he, and that Solomon said of wisdom, "Length of days is in her right hand, and in her left hand riches and honor."

Educate the people, of all classes and conditions, to say with the distinguished Dr. Parkes, of England: "It has been proved over and over again, that nothing is so costly in all ways as disease, and that nothing is so remunerative as the outlay which augments health, and in so doing augments the value of the work done."

Finally, your "Code of Ethics"—that grand yet simple code of laws, replete with lessons of self-abnegation, philanthropy and exalted dignity, which to the honorable physician is as sacred as the Bible to the Christian—makes it your duty to enlighten your communities upon questions of

HYGIENE.

It is your duty to constantly, zealously and fearlessly preach the gospel of health. Have you done it? If not, you have been recreant to the solemn obligation which you assumed upon entering the ranks of the medical profession.

Did you ever know an honest, learned, philanthropic physician to pretend to disdain the science of hygiene? Physicians may differ conscientiously upon some of the principles of this science, and may disapprove of some regulations of health organizations, but no man but the monomaniac, the knave or the fool, can attempt to deride the science of preventive medicine. Show me the man who scoffs at hygiene and I will demonstrate to you that he is one whose soul dances to the jingles of dollars wrung from diseased and suffering people; a man into whose heart the warm, generous glow of philanthropy never shed its benign rays; whose greed for gold has so dwarfed his soul that you might shake it in a mustard seed for a thousand years without expanding the limits of seed or soul. Look over the long list of men who have assiduously cultivated this science. Who are they? What is their reputation for learning, integrity, philanthropy?

We often hear it said that a new era is dawning over the science of medicine; that of late the physician is impressed with the fact that it is his duty to prevent as well as cure diseases. This statement emanates only from the man who is ignorant of the history of medicine. It is true that in its earlier history medicine was devoted to the cure or relief of disease. Experience soon taught the physician that it was often an easier task to prevent certain diseases than to cure them when developed in the human system. From decade to decade physicians have more and more thoroughly appreciated the inter-relationships which exist between preventive and remedial medicine. However long the time ere this truth was fully appreciated, it has long been fixed as one of the certainties of our science that no man can be regarded as a cultivated, successful physician who is not a learned sanitarian, and no man can be a learned sanitarian except he be an accomplished physician. The physician who rests satisfied with being a mere physic-giver knows nothing of the genius or grandeur of medicine. To intelligently understand the laws of disease he must know the laws of health. The physician's first knowledge must be of health in order that he may definitely ascertain the shades and lines of departure therefrom, and the better understand how to recall the diseased body to its normal healthfulness. "Medicine is a science which aims at the preservation of health, the cure of diseases and the physical perfection of man." This is the only definition which does justice to our honorable profession. To the resplendent honor of man and the glory of his Maker, be it remembered that the science of hygiene is the self-sacrificing, free gift of the medical profession. God forbid that I should attempt to belittle or deride the curative or remedial part of medicine. I have the most unbounded scorn and contempt for the physician who would do so. I do contend, however, that the physician who devotes his time and talents to the acquisition of knowledge, of the disturbing forces of health, and who is thereby able to remove or limit the operation of these forces ere they cast a burden of disease and death upon mankind, is treading the most exalted and beneficent walk in the royal profession of medicine. This science called hygiene has commanded the admiration and enlisted the active, soulful efforts of the highest, brightest and purest members of our profession. These are the men who are enthusiasts upon the subject of hygiene. These men have experienced the luxury of doing good, and they are known to the honor and glory of the grand science of medicine. The cold, inhuman philosophy of "the survival of the fittest" finds no response in the heart of the true physician. The whole life of man under past and present surroundings has been, and is, a fight for life. His power to cope with disease and death is founded upon an intelligent conception of the laws of health, and the conservation of the vital forces with which he is endowed. It is your exalted province to disseminate this knowledge. Be true to the highest mission of your life-work. Teach man of the high purposes and possibilities of life. Teach your brother man to appreciate the fact that he was created for health, not disease; for long life, not premature death. The hope of saving any number of human lives is enough to fire the enthusiasm of the brightest, noblest, best of God's creatures. Enter this field of life-saving. Press onward and upward. Be not dismayed by the sneerings of the pessimist who will call you a fanatic, and when well-nigh borne down by doubts from without and from within, never hesitate; remember that

——"our doubts are traitors,
And make us lose the good we oft might win
By fearing to attempt."

